# The impact of imprisonment on individuals’ mental health and society reintegration: study protocol

**DOI:** 10.1186/s40359-023-01252-w

**Published:** 2023-07-25

**Authors:** Olga Cunha, Andreia de Castro Rodrigues, Sónia Caridade, Ana Rita Dias, Telma Catarina Almeida, Ana Rita Cruz, Maria Manuela Peixoto

**Affiliations:** 1 HEI-Lab: Digital Human-Environment Interaction Labs, Lusófona University, Porto, Portugal; 2 Faculty of Psychology, Education, and Sports, Lusófona University of Porto, Rua Augusto Rosa, 24, Porto, 4000-098 Portugal; 3 William James Center for Research, ISPA – Instituto Universitário de Ciências Psicológicas, Sociais e da Vida, Lisbon, Portugal; 4 Research Center of Psychology, School of Psychology, Minho University, Braga, Portugal; 5 Egas Moniz Center for Interdisciplinary Research (CiiEM), Egas Moniz School of Health & Science, LabPSI – Laboratório de Psicologia Egas Moniz, Almada, Portugal; 6 HEI-Lab: Digital Human-Environment Interaction Labs, Lusófona University, Lisbon, Portugal; 7 Center for Psychology at University of Porto, Porto, Portugal

**Keywords:** Mental health, Prison, Conditional release, Social reintegration

## Abstract

**Background:**

Prison sentences are a particular type of penalty that aim to reintegrate individuals into society. Nonetheless, research suggests that prison sentences have a null or a criminogenic effect on recidivism and a critical impact on inmates’ mental health, negatively interfering with their successful reintegration into society and recidivism. Prevalence rates of mental health disorders among individuals who commit crimes are high, but little is known about how incarceration perpetuates and/or worsens mental health symptoms. In the Portuguese context, no studies focused on understanding the impact of imprisonment on prisoners’ mental health. Thus, this project aims to understand incarceration’s mental health and well-being impact on male and female individuals convicted to prison, both while incarcerated and after release.

**Methods:**

The study will follow a quantitative cross-sectional design of male and female individuals in prison and parole, aiming to assess different samples at different moments of the prison sentence. It will also follow a longitudinal design in a subsample of male and female individuals sentenced to prison and on parole who will be followed for one year.

**Discussion:**

This study intends to have a meaningful impact on the understanding of imprisonment effects, giving important clues for developing and implementing evidence-based prevention and intervention strategies to address prisoners’ and ex-prisoners’ mental health and improve their ability to successfully reintegrate into society and reduce recidivism.

## Background

In many contexts worldwide, penal sanctions aim to reintegrate individuals into society, and prison sentences are one such sanction. However, research has found that imprisonment has a weak or null effect, or even a criminogenic effect on recidivism rates [1], as well as a detrimental impact on inmates’ mental health and well-being [2–5], which, in turn, may significantly impact their successful reintegration into society and reoffending/recidivism rates [5]. This impact on individuals’ mental health poses considerable challenges to the Criminal Justice System (CJS), including the higher use of prison healthcare services and increasing institutional costs [4].

Psychopathology among prisoners has been linked to violence, self-harm, suicide, victimization, and reduced willingness or ability of individuals to participate in daily activities and prison programs, which may impact their well-being and rehabilitation [2, 4, 5]. In addition, research points to considerable rates of mental health disorders among those who committed crimes and were imprisoned and higher comorbidity between mental illness and substance misuse [2, 3, 5]. From a trauma deprivation perspective, prison can have even more severe detrimental effects on women’s mental health than men’s [6]. Also, compared to their male counterparts, women have higher rates of psychiatric disorders (e.g., depression and drug dependence) [7].

Nonetheless, little attention has been paid to how incarceration fosters the onset of psychological symptoms or perpetuates and worsens previous psychological symptoms. A key issue in this field is the direction of causality for the high prevalence of mental disorders in prisons - whether the increased rates are caused by prison or imported into prison [3, 5, 7]. This debate is rooted in a theoretical framework considering importation versus deprivation models. From the importation approach, different studies suggested that pre-prison adversities (e.g., illiteracy, child abuse, homelessness, mental illness) contribute to subsequent mental illness among some prisoners. From the deprivation approach, the research found that some prisoners develop a mental illness due to the prison environment [7]. Although how people react to imprisonment varies from person to person, incarceration is associated with poor mental health outcomes [2, 5].

Several personal risk factors have been identified, like female gender, White race, socioeconomic and academic/occupational deficiencies, traumatic experiences, reduced social support, coping style, substance use, and brain injuries. In addition, pre-existing mental illness may worsen mental health in prison [4]. Studies showed that imprisonment has more iatrogenic than deterrent effects on offenders [2], considering that individuals face different barriers and constraints while in prison. In addition to personal variables, factors related to the prison environment and aspects of the correctional climate negatively impact prisoners’ mental health. The prison environment can be inherently damaging to mental health due to the consequent disconnection from family, society, and social support, loss of autonomy, diminished meaning and purpose of life, fear of victimization, increased boredom, the unpredictability of surroundings, overcrowding and punitiveness, experiencing and witnessing violence, negative staff-prisoner interaction, and other aversive experiences [2, 5, 6, 8, 9].

The effect of incarceration also depends on other factors, such as the time served in prison and the period related to imprisonment and release. For instance, the first weeks of imprisonment and the period following release are associated with a higher risk of suicide [10]. Longer sentences are also related to increased healthcare needs [11].

In line with the transactionist theory of prisoners’ adjustment [12], environmental characteristics, in interaction with individual ones, may act as a source of pressure for prisoners’ behaviour. Prisoners seem to import trauma’s negative and detrimental effects into prison – importation - but when imprisoned, it is common for prisoners to experience additional traumas (e.g., violence) – deprivation [5]. Trauma adverse effects are cumulative; thus, prisoners are at-risk for developing or aggravating mental illness. In this sense, mental illness in prison could be attributable to importation and deprivation. Incarceration can also lead to post-incarceration syndrome, a syndrome like posttraumatic stress disorder (PTSD); even after serving the prison sentence, many individuals continue to suffer its mental effects [13]. Some effects may include institutionalized personality traits, such as distrusting others or difficulty maintaining relationships, social-sensory disorientation, and social-temporal alienation.

According to the General Strain Theory (GST), individuals may experience three types of strain: (i) the failure to achieve positively valued goals, (ii) the removal of positively valued stimuli, and (iii) the presence of negative stimuli [14]. These stressors lead to negative emotions like anger and frustration. Individuals without adequate coping mechanisms to relieve these feelings may turn to criminal pathways. Poor mental health may act as a strain that includes all three domains of stressors [15]: when an individual has poor mental health, (s)he may not be able to achieve desired goals; poor mental health may lead to the loss of positively valued stimuli; and mental health problems are noxious stimuli that may cause the person discomfort [15]. All these conditions make the individual more prone to use maladaptive behaviours while imprisoned or after release.

Although the effects of imprisonment on inmates’ mental health have been documented in international research, in Portugal, as far as we know, there are no studies focused on examining the effects of imprisonment on inmates’ mental health, both during incarceration and after release. The current project aims to examine the mental health status of male and female individuals convicted of prison sentences, both while incarcerated and after release. The following specific aims were defined: (a) to assess the impact of incarceration on male and female prisoners’ mental health; (b) to examine the impact of sentence length and period on male and female prisoners’ mental health; (c) to examine changes in prisoners’ mental health symptoms during incarceration and after release; (d) to examine differences between male and female prisoners’ mental health during the time they were serving their sentence and while in parole; (e) to analyse the factors/variables (sociodemographic, criminological, personal, prison environment) that are linked to mental health impairments during imprisonment, in men and women; (f) to analyse the possible mediation and/or moderation effects of the personal and prison environment factors on male and female prisoners’ mental health; (g) to examine the impact of mental health on prisoners’ prison misconduct; and (h) to examine the impact of mental health on male and female individuals’ adjustment to the community after release.

## Methods/design

This project includes two studies with two different methodological designs: a cross-sectional design (study 1) and a longitudinal design (study 2).

### Participants

#### Study 1

The sample was determined according to the universe of male (N = 11,507) and female individuals (N = 901) in prison, and male (N = 2632) and female (N = 196) individuals on conditional release and the following criteria: significance (i.e., the number of sample’s effective) and representativeness (i.e., sample’s quality guaranteed by the sampling method). Sample significance was calculated using the Krejcie and Morgan (1970) formula. Thus, a minimum of 375 male and 269 female participants in prison and 338 male and 132 female participants on conditional release will be recruited.

#### Study 2

As a longitudinal study, it will be based on a convenience sample of individuals in prison and in conditional release. We expect to recruit a minimum of 100 men and 80 women in prison and 60 men and 40 women in conditional release. A priori power calculations revealed that the sample size is adequate to conduct the analyses (effect size = 0.30, power = 0.95, number of latent variables = 1, number of observed variables = 5, *N* = 100) [16].

### Procedures

After obtaining approval from the Host Institution’s Ethics Committee, authorization to recruit and assess male and female individuals who are in prison and conditional release (studies 1 and 2) will be obtained from the General Directorate of Reintegration and Prison Services–Ministry of Justice (DGRSP-MJ). Then, different national Prisons and Social Reintegration Teams (from the North to the South of the country) will be contacted to define the data collection procedures for studies 1 and 2.

All the individuals will sign an informed consent in which the main objectives of the research, the voluntary and anonymous nature of their participation, and the fact that no risks or benefits (e.g., financial, legal, or others) are expected from participating will be explained. Data will be collected in a paper-and-pencil format (i.e., presential).

#### Study 1

Study 1 uses a cross-sectional design of male and female individuals in prisons and conditional release. Inclusion criteria included adult male and female individuals in prison and on conditional release of Portuguese nationality and older than 18 years. Since this study aims to assess different samples at different moments of the prison sentence, five matched groups of male and female individuals convicted of prison will be assessed: a group of individuals at the beginning of the prison sentence (1–2 weeks at a maximum); a group of individuals six months after the beginning of the prison sentence; a group of individuals at the end of the prison sentence; a group of individuals 1–2 weeks after the prison release; and a group of individuals one year after prison release. To ensure the sample’s representativeness, information about individuals older than 18, distributed by sex and age from the different national Prisons and Social Reintegration Teams, will be requested from the DGRSP-MJ. Participants will be selected randomly.

#### Study 2

Study 2 uses a longitudinal design in a subsample of adult male and female individuals in prisons and conditional release. Inclusion criteria include (a) adult male and female individuals (i.e., 18 years or more); and (b) individuals who recently entered prison (1–2 weeks in maximum) and individuals who were recently released from prison (1–2 weeks in maximum).

Male and female individuals who participated in study 1 and who were integrated into the group of individuals at the beginning of the prison sentence (1–2 weeks at a maximum) and into the group of individuals 1–2 weeks (at a maximum) after the prison release will be invited to participate in the longitudinal study. Confidentiality of data will be guaranteed. However, an alphanumeric code will be attributed to each participant and maintained until the last data collection moment (12 months) to match the participants and anonymize the participation. At the end of the data collection, all the codes will be deleted, and no personal information will be maintained.

As it will be a longitudinal study, after the baseline assessment, individuals will be followed and assessed at routine intervals of three months for one year - three months, six months, nine months, and one year.

### Measures

***Sociodemographic and Juridical-Penal Questionnaire*** will be used to collect data on sociodemographic (e.g., age, sex, marital status, educational level, socioeconomic level, etc.) and juridical-penal variables (e.g., sentence length, crime committed, recidivism, etc.).

***Adverse Childhood Experiences Scale*** (ACEs) [17, 18] is a brief self-report measure with ten items for assessing ten types of childhood adversities: physical, verbal, and sexual abuse, physical and emotional neglect, exposure to domestic violence, alcoholic parent, a family member in jail, a family member with a mental disorder, and parents’ divorce.

***Benevolent Childhood Experiences Scale*** (BCEs) [19, 20] is a 10-item self-report tool for assessing supportive and positive experiences from birth to 18 years of age.

***Alcohol, Smoking, and Substance Involvement Screening Test*** (ASSIST) [21, 22] is a screening tool for assessing the risk level for alcohol, smoking, and other substance abuse and dependence and the main consequences of alcohol, smoking, and other substance abuse.

***Prison Environment Inventory*** (PEI) [12, 23] is a 48-items self-report tool for assessing the prisoner’s perception of the prison environment, including eight dimensions: privacy, safety, structure, support, emotional feedback, social stimulation, activity, and freedom.

***Depression, Anxiety, and Stress Scales − 21*** (DASS-21) [24, 25] is a 21-items self-report measure for assessing depression, anxiety, and stress symptoms. Higher scores indicate more severe symptomatology.

***Posttraumatic Stress Disorder Checklist for DSM-5*** (PCL-5) [26, 27] is a self-report measure with 20 items that allow it to assess the 20 DSM-5 PTSD symptoms. Higher scores indicate more severe symptomatology.

***Suicide Behaviours Questionnaire-Revised*** (SBQ-R) [28, 29] is a very brief screening of suicidal behaviours, with higher scores indicating more frequent suicidal behaviours and greater suicide risk.

***Difficulties in Emotion Regulation Scale-Short Form*** (DERS-SF) [30, 31] is a 18-items self-report assessment tool that assesses six dimensions of difficulties in emotion regulation: awareness; clarity; goals; impulse; non-acceptance; and strategies. Greater scores indicate more severe difficulties in the emotion regulation process and strategies.

***Buss-Perry Aggression Questionnaire – Short Form*** (BPAQ-SF) [32, 33] is a very brief self-report instrument with 12 items for assessing aggression through four main dimensions: physical aggression, verbal aggression, anger, and hostility. Higher scores suggest greater levels of aggressiveness.

***Satisfaction with Life Scale*** (SWLS) [34, 35] is a brief measure with five items developed for assessing global and overall satisfaction with one’s life. Greater scores describe a greater perception of satisfaction with life.

***Social Support Satisfaction Scale*** (ESSS) [36] is a 15-items measure for assessing satisfaction with social support, including with family, friends, and community. Higher scores on the scale indicate greater satisfaction with social support.

***Brief Resilience Scale*** (BRS) [37] is a brief self-report tool with six items for assessing resilience and recovering from stress. Higher scores suggest a greater ability to recover from stress and be resilient.

***Delinquency Questionnaire*** (D-CRIM) [38] is a 12-items self-report measure to assess criminal behaviours (violent and non-violent) that occurred in the last 12 months or lifetime.

***Social Network Index*** (SNI) [39] is an assessment tool for measuring the degree of participation of an individual in 12 different types of social relationships and networks (e.g., intimate partner, parents, children, family members, neighbours, friends, and workmates).

***Individual files*** of participants will also be examined to extract information related to criminological variables (e.g., length of the sentence), institutional misconduct (e.g., number of infractions), mental health services use, medication, and number of visits in prison.

Information regarding the different prisons will be collected, such as prison size, security level, and overcrowding.

### Data analysis

#### Study 1

Descriptive statistics using the IBM SPSS version 28.0 will be conducted to characterize the sample and the main variables. Univariate, bivariate, and multivariate analysis will be conducted, along with mediation and moderation analysis using PROCESS macro 4.2 for IBM SPSS software.

#### Study 2

Mixed-effects models will be performed using IBM SPSS version 28.0, combining two levels to a single framework, with Level 1 for repeated measurements nested within Level 2. Longitudinal Structural Equation Modelling will also be used to examine causal inferences through moderation and mediation effects using AMOS IBM SPSS.

## Discussion

Despite international decarceration movements and the decrease in male incarceration rates in the last years, in Portugal, 12,408 individuals were in prison on 15 April 2023. Incarceration’s personal, judicial, societal, and financial costs are burdensome. Each prisoner costs the Portuguese State €49 per day on average. According to international data, this figure doubles if mental health expenses are added to daily expenses. However, the costs of not preventing or intervening in prisoners’ mental health problems can be even more damaging for individuals and the entire society than not treating them. Incarceration is definitively linked to poor health, and upon release from prison, many individuals experience difficulties in maintaining good health [6]. In addition, the combination of better mental health in prison and increases in mental health post-release is associated with a reduced likelihood of reoffending [6]. Consequently, reductions in recidivism lead to reductions in the prison population [40]. Considering the costs of imprisonment, reduced recidivism will lead to financial impacts on the CJS. There is also evidence of cost-effectiveness in providing mental health interventions and treatment during incarceration and post-incarceration [41]. Providing mental health services and programs in prison and after release is a legal (societal and humanitarian) imperative and a way to improve individuals’ skills and coping strategies to increase their successful reintegration into society after prison release (and, therefore, decrease reoffending and recidivism rates).

With this project, we expect to demonstrate that imprisonment has a pernicious effect on inmates’ mental health and well-being that lasts even after prison release, which, in turn, might significantly impact individuals’ reintegration into society and reoffending. However, we expect to find changes in mental health adjustment during the prison sentence and the conditional release. We expect to expand the current knowledge on the effects of incarceration using an innovative design. We also intend to challenge the prevailing view about prison sentences and their impact on individuals’ rehabilitation, claiming attention to the damaging effects of such penalties, both to the individual and the entire society. This study will also give us important clues for developing and implementing more effective and evidence-based prevention and intervention strategies to address mental health disorders among prisoners and ex-prisoners and, therefore, improve their ability to successfully reintegrate into society and reduce recidivism.

Improving the understanding of inmates’ mental health conditions and how imprisonment impacts mental health and well-being is relevant, considering pre-imprisonment factors and being able to develop effective strategies to prevent and reduce its occurrence. Specifically, results from this study will inform the development of prevention and intervention efforts to identify those at risk early and improve their abilities and skills to successfully adapt to imprisonment and reintegration into society, breaking the cycle of release-recidivism-reimprisonment. Finally, this project is aligned with the United Nations Sustainable Development Goals (SDG) as it will contribute to ensuring healthy lives and promoting well-being among prisoners, reducing inequalities for those affected by imprisonment, and promoting peaceful and inclusive societies for sustainable development, providing access to justice for all, and building effective accountable and inclusive institutions.

## Data Availability

Available upon request to the corresponding author.

## Abbreviations

ACEs: Adverse Childhood Experiences Scale
ASSIST: Alcohol, Smoking and Substance Involvement Screening Test
BCEs: Benevolent Childhood Experiences Scale
BPAQ-SF: Buss-Perry Aggression Questionnaire ? Short Form
BRS: Brief Resilience Scale
CJS: Criminal Justice System
DASS-21: Depression, Anxiety and Stress Scales - 21
D-CRIM: Delinquency Questionnaire
DERS-SF: Difficulties in Emotion Regulation Scale-Short Form
DGRSP-MJ: General Directorate of Reintegration and Prison Services?Ministry of Justice
ESSS: Social Support Satisfaction Scale
GST: General Strain Theory
PCL-5: Posttraumatic Stress Disorder Checklist for DSM-5
PEI: Prison Environment Inventory
PTSD: Posttraumatic stress disorder
SBQ-R: Suicide Behaviours Questionnaire-Revised
SDG: Sustainable Development Goals
SNI: Social Network Index
SWLS: Satisfaction with Life Scale
